# Editorial: Emerging SARS-CoV-2 variants: genomic variations, transmission, pathogenesis, clinical impact, and interventions, volume II

**DOI:** 10.3389/fmed.2023.1215309

**Published:** 2023-06-28

**Authors:** Pragya D. Yadav, Deepak Y. Patil, Sanjay Kumar, Eric Bergeron, Sergio E. Rodriguez

**Affiliations:** ^1^Indian Council of Medical Research-National Institute of Virology, Pune, Maharashtra, India; ^2^Department of Neurosurgery, Command Hospital (Southern Command), Armed Forces Medical College (AFMC), Pune, India; ^3^Centers for Disease Control and Prevention (CDC), Atlanta, GA, United States; ^4^Galveston National Laboratory, Department of Microbiology and Immunology, University of Texas Medical Branch, Galveston, TX, United States

**Keywords:** genomic variations, transmission, pathogenesis, clinical impact, intervention

## Summary

This Research Topic has focused on subjects such as tracking emerging SARS-CoV-2 variants, detection, isolation, and genomic characterization of emerging variants, transmission, pathogenesis, clinical effects of variants, assessment of COVID-19 vaccination and treatment effectiveness, comparative analysis of SARS-CoV-2 genomic data, and other public health intervention measures. The Research Topic featured 27 articles highlighting the emergence of Omicron variants and their sub-lineages across the globe and their clinical presentations, specifically asymptomatic infections, COVID-19-associated liver injury, and comorbidities such as hypertension, diabetes, and bronchitis. Additionally, a few studies have reported the efficacy of therapeutic drugs in reducing viral load and the significance of vaccination and a booster dose against Omicron variants. Furthermore, the studies on genomic surveillance and evolutionary analysis have demonstrated the emergence of Omicron and its sub-lineages and their characteristic mutations. All these in-depth studies have explored various elements of Omicron, resulting in a comprehensive understanding of this variant.

## Background

The ongoing emergence of SARS-CoV-2 variants offers a challenge for long-term COVID-19 control. The severity of COVID-19 has been successfully prevented by vaccination. However, SARS-CoV-2 genomic mutations leading to immune escape and higher transmissibility increase the severity of COVID-19 by either increasing virulence or decreasing vaccine efficacy. The severity of COVID-19 disease can range from asymptomatic to mild, moderate, severe, and critical. The likelihood of developing a severe illness increases with the number of underlying medical disorders, and it is more common in older people and those who have pre-existing diseases. A timely clinical characterization of SARS-CoV-2 infections is necessary to aid policy-making; however, data on specific COVID-19 cases and the associated SARS-CoV-2 variants is only accessible in a few situations.

In addition, the SARS-CoV-2 virus has undergone changes through natural evolution over time. Compared with non-variant viruses, mutations might cause immune escape, enhance or decrease virulence or transmissibility, or impair the response to treatments. Specific mutations and changes in amino acids in variant spike and non-spike proteins may modify tissue tropism or enhance virulence, which may have an effect on clinical presentation. To adapt to the host, the Omicron variant uses a different approach than the Delta and other variants, leading to distinct cell entrance paths and clinical symptoms. As evidenced by many studies, Omicron has a higher transmissibility than the earlier SARS-CoV-2 variants. However, it has proven challenging to definitively pinpoint specific mutations as the cause of increased virulence or altered tissue tropism. New variants will eventually appear, and it is anticipated that the transmissibility and virulence will evolve.

With the emergence of Omicron, it quickly became apparent that vaccine effectiveness was lower for Delta because Omicron can still produce symptomatic infections in individuals who have had their initial immunisations. Boosters offer very high levels of defence against the possibility of contracting a serious illness, hospitalization, and mortality. The COVID-19 vaccine offers excellent protection against hospitalization, especially after three doses. A mild symptomatic infection can progress to a more serious illness in some people. Vaccines can stop these mild diseases from occurring and are effective in preventing serious illness, hospitalization, and death.

Therefore, understanding the clinical and genomic evolution of SARS-CoV-2 is crucial for early diagnosis and exploring therapeutics and vaccine efficacy to lower morbidity and mortality.

## Clinical impact and interventions

Age, vaccination status, and variants of concern all influence the clinical aspects of COVID-19, which appear in different ways in terms of frequency and severity. Individuals with more severe symptoms are frequently overrepresented in published reports, and these symptoms may vary throughout care settings between different age groups and vaccination statuses. At the beginning of the infection, there may be no symptoms, but as the condition progresses, symptoms may start to appear. Additionally, it has been observed that patients who had received the COVID-19 vaccine and were admitted to the hospital during the Omicron variant surge had fewer severe illnesses than those who had not received the vaccine, and they were also less likely to be sent to intensive care. Various researchers across the globe have reported the emergence of different Omicron sub-variants, immune escape, and unusual clinical presentations of COVID-19 cases.

In this Research Topic, Kouamen et al. studied the features of cases infected with the Omicron BA.4 and BA.5 variants in France. The likelihood of hospitalization was approximately 17 times higher in cases with at least one risk factor than in cases with none. The BA.4 and BA.5 variants showed no notable clinical manifestation globally despite their prolonged duration, changing symptoms, and probable immune escape (Kouamen et al.). Peng et al. reported an Omicron BA.2 case presenting with mild acute respiratory distress syndrome. This case showed an improved inflammatory index and a lowered oxygen index with multiple a treatment regimen. The COVID-19 quick antigen test performed at home may supplement the detection techniques now in use. The COVID-19 vaccine booster dose might be advantageous in the event of newly emerging Omicron sub-lineages (Peng et al.). Additionally, Zhang et al reported COVID-19 cases (*n* = 169) infected with Omicron and hospitalized in Suzhou, China. The median time from the start of the disease to hospitalization was 2 days with the three main comorbidities diabetes, bronchitis, and hypertension. A sizeable part of the population was made up of asymptomatic individuals. There were no documented cases of seriously sick or deceased patients. According to the study's findings, a booster dose or complete immunization is required to protect against the viremia of the omicron variant (Zhang, Chen, et al.).

Patients with or without pre-existing liver illness frequently experience COVID-19-associated liver damage, which is linked to a more severe course of the infection and other consequences, including mortality. Zhang, Zhao, et al. observed liver dysfunctions in COVID-19 cases. The liver damage in the cases infected with Omicron was less severe than those infected with B.1 and Delta. The findings suggested that the viremic impact of Omicron tended to be minor, while the liver damage it induced was less than that of the earlier circulating variations (Zhang, Zhao, et al.). Additionally, Chen et al. reported severe acute hepatitis in a child with BA.2.38 infection in China. This case emphasizes the possible risk of acute liver illness in children with mild COVID disease. Clinicians can benefit greatly from the concept of differential diagnosis (Chen et al.). Influenza and COVID-19 both induce respiratory diseases and have a high mortality rate. Individuals may have varying degrees of sickness from COVID-19 and influenza. Recently, Zhang, Huang et al. compared COVID-19 cases with B.1 and Delta infections with mild seasonal influenza. According to the data collected during hospitalization, there is a stronger clinical link between patients with influenza and those who are infected with B.1 than those infected with delta. The COVID-19 pandemic has highlighted the urgent need for preventive and sufficient immunizations against the flu and COVID-19 along with improved treatment regimens (Zhang, Huang, et al.). He et al. identified the risk factors, i.e., eosinophil count, neutrophil to lymphocyte ratio, albumin levels, and CD4/CD8 ratio, associated with prolonged viral shedding among mild Omicron cases.

The abrupt rise in COVID-19 cases across the globe is suggestive of the emergence of variants with selection advantages. Selvavinayagam et al. studied the demography, clinical presentation, and markers of adults hospitalized with COVID-19 in Chennai, India. The following mutations were particular to BA.1.2: A27S, D405N, L24S, P25del, P26del, R408S, T376A, T19I, and V213G. Increased probabilities of recovering or having an asymptomatic illness were independently correlated with the number of vaccination doses received. This implies that the new mutations described here may have a major influence on the disease course, clinical, and epidemiological features of the virus (Selvavinayagam et al.). In addition, Lavania et al. examined four cases of a severe multisystem hyperinflammatory syndrome in children between the ages of 11 and 15 that occurred during the SARS-CoV-2 epidemic and were later determined to be brought on by Echovirus-18 (Enterovirus). A prompt, efficient, and potentially life-saving course of treatment depends on an accurate and early diagnosis.

## Vaccine efficacy and therapeutics

The COVID-19 pandemic has had a serious impact on humanity as a whole and presents a significant challenge to the public health systems of the afflicted nations. During the early phase of the pandemic, many research teams at biomedical universities, governmental organizations, and private biotech corporations have intensified and focused their studies on finding and assessing potential COVID-19 vaccine candidates and therapeutics. With these efforts, many vaccine candidates and antivirals have been developed and approved under Emergency User Authorization (EUA); however, COVID-19 remains untreated. The main therapies for the illness were respiratory therapy, antivirals, and anti-inflammatories. Additionally, antibody therapies are currently a very active and crucial component of the treatment for SARS-CoV-2 infection. Several treatment alternatives, including novel antivirals, monoclonal antibodies, immunoglobulins, and convalescent plasma therapy are being explored in ongoing trials. For the purpose of developing intervention measures, it is crucial to comprehend how factors such as prior SARS-CoV-2 infection, monoclonal antibody therapy, and COVID-19 vaccination-induced immunity affect the probability of Omicron infection and serious outcomes. According to the studies, getting vaccinated against COVID-19, including a booster dose, is still essential for reducing the chance of developing serious illness. Numerous studies have discussed the effectiveness of COVID-19 treatments and vaccines in this Research Topic.

In this Research Topic, Paxlovid's effectiveness in treating older people with Omicron was reported by Zhong et al. Paxlovid has been found to dramatically lower the virus-shedding duration in older people with Omicron compared with the control group. Uncertainty exists regarding how the medications nirmatrelvir and ritonavir affect the shedding of SARS-CoV-2. Kim et al. demonstrated the effectiveness of nirmatrelvir/ritonavir treatment in decreasing viral loads in Omicron cases; the duration of virus shedding was not shortened.

In Merida, Mexico, Puerta-Guardo et al. investigated the IgG antibody response in individuals vaccinated with either a single dose of the Adv5-nCoV or BNT162b2 vaccine. More than 25 days after vaccination, all of these recipients showed an overall IgG seroconversion. Surprisingly, antibodies against the N protein were found in more than 50% of vaccine recipients who had never previously contracted COVID-19 (Puerta-Guardo et al.).

In addition to examining the impact of rheumatoid arthritis (RA) medications on vaccine immunogenicity, Zhao et al. investigated the immune response in RA patients with a third dose of inactivated vaccine. After the third vaccination, NAb titers were considerably lower in RA patients than in healthy controls (HCs), and the positive NAb rate in the HC group was 90.4% compared with 80.18% in RA patients, a significant difference. This investigation will aid in assessing the effectiveness of booster vaccination among RA cases (Zhao et al.).

A number of variants of concern (VOC) have emerged as a result of uncontrolled transmission of the SARS-CoV-2 coronavirus. In a cohort of university staff members and students who were COVID-19-naive and had received two or three doses of mRNA vaccination, Dai et al. assessed the presence of antibodies against the N protein to determine both breakthrough infections with and without symptoms. Four breakthrough infections (BTIs) caused by Delta and Omicron were recorded among the participants (4.7%). Neutralizing antibodies against Delta or Omicron had increased by more than fourfold in two of the three symptomatic BTIs, as well as during the reinfection. The study's conclusions highlight the use of anti-nucleocapsid antibody for testing the post-vaccination period (Dai et al.).

## Genomic analysis of SARS-CoV-2

Countries around the world are preparing for COVID-19 to transition from a pandemic to an endemic phase, but the advent of novel SARS-CoV-2 strains has made the situation worse. SARS-CoV-2 is perfectly adapted to its human host and newly emerged variants led to different waves of COVID-19. Researchers across the globe have been carrying out genomic surveillance to determine the circulating SARS-CoV-2 variants. They are continuously accumulating SARS-CoV-2 sequences and analyzing the differences between these sequences from different geographical locations. The data on the transmission of variants and modifications to the genetic makeup of SARS-CoV-2 variants are utilized collectively to evaluate how variants might affect public health. Thus, it is important to undertake studies that will illuminate the evolutionary pattern of SARS-CoV-2 globally.

In this Research Topic, Yu et al. reported that distinct clades predominated over the COVID-19 waves in Malaysia, with the L and O clades dominating the first two waves and the GRA clade gradually being replaced by the G, GH, and GK clades in subsequent waves. Recombination events have been described in the *Coronaviridae* family (Yu et al.). Silva et al. who discovered the BA.1.1 and BA.2.23 recombination event in Brazil, characterized four novel mutations. Additionally, they identified a new lineage, XAG, clustered in a monophyletic clade (Silva et al.).

In November 2021, researchers discovered Omicron, a novel SARS-CoV-2 variant. Sharma RP et al. carried out genomic analysis of COVID-19 cases in Rajasthan, India to determine the relationship between illness severity and genomic profile. Most cases were asymptomatic followed by mild disease and significant symptoms and two had serious disease that required hospitalization; one patient died, while the other 97% made a full recovery (Sharma et al.). The clinical presentation and genomic characterization of COVID-19 cases in Uttar Pradesh, India between January 1 and February 24, 2022, were also examined by Zaman et al. BA.2 was more prevalent than BA.1 in eastern Uttar Pradesh, with distinctive spike mutations in the BA.1.1 and BA.2.1 strains. Dhanasooraj et al. examined the RBD region of SARS-CoV-2 using in-house methods in Kerala, India from March 2021 to May 2022. The outcomes were largely comparable with those from other regions of India and other nations at the time (Dhanasooraj et al.).

According to Romano et al., AY.99.2 most likely appeared between the end of April and the beginning of May 2021 in Brazil, a few weeks after the detection of B.1.617.2, and quickly spread to other nations. In addition, da Silva discussed how the introduction of novel SARS-CoV-2 omicron sub-variants raises questions about when the epidemic will be over. The connection of the ORF3A protein and subcellular sites was investigated by Cruz-Cosme et al. through a thorough mutagenesis analysis. The mutations in the YXX motif and double glycine (diG) region, which are necessary for protein export, showed the same phenotype. According to structural investigations, the diG motif connects to the Golgi apparatus and aids a type II turn among the antiparallel 4 and 5 sheets. To reduce the detrimental effect of mutations on the effectiveness of genome targeting, Bei et al. showed how to evaluate, test, and improve sequencing and detection procedures, using SARS-CoV-2 as an illustration.

Angiotensin-converting enzyme-2 is part of the spike protein, which helps the virus enter the human cell. According to Mahase et al., D350 mutations in ACE2 have the most stabilizing effects on the protein. They also discovered genetic changes in ACE2 in African Americans and Latino Americans, with both populations having an impact on ACE2 complex stability. Open-source software was used by Ruiz et al. to analyze the possible immune evasion of the viruses and the interaction of the ACE2 receptor. The Omicron variant seems to be better at thwarting immunological reactions (Ruiz et al.). A low percentage of sequenced samples, various variants connected to several reintroductions, and a rise in the frequency of mutation are just a few of the findings for Latin America that Molina-Mora et al. showed are consistent with worldwide data. In addition, 83 lineages, including Gamma, Mu, and Lambda (Molina-Mora et al.), have flourished locally with nation-specific enrichments. Additionally, Mahilkar et al. discussed the mechanisms behind virus-host interaction, new variants, and noteworthy mutations and their potential effects on diagnosis, clinical presentation, and case management.

## Conclusion

The success of the global genomic surveillance of SARS-CoV-2 has helped in the development of new tools and technologies for tracking and predicting the genomic evolution and spread of emerging variants. These advancements will continue to be invaluable in future outbreaks and pandemics. Many studies have shown that immunity to COVID-19 may last for at least several months, but it is still unclear how long it will last. Ongoing research is needed to determine the duration of immunity and the risk of reinfection over time. The duration and strength of immunity may vary depending on factors such as age, severity of illness, and individual immune response. Therefore, research is needed to determine the long-term effectiveness of vaccines and natural immunity. The extent and timing of subsequent waves will be determined by the transmissibility and immune-evasiveness of emerging SARS-CoV-2 variants. The reduction of COVID-19-related morbidity and mortality depends on sustained SARS-CoV-2 surveillance efforts to assess the effects of interventions.

## Author contributions

PY and DP wrote the first draft. The final manuscript has been reviewed and approved by all the authors.

